# CO_2_/CH_4_ and He/N_2_ Separation Properties and Water Permeability Valuation of Mixed Matrix MWCNTs-Based Cellulose Acetate Flat Sheet Membranes: A Study of the Optimization of the Filler Material Dispersion Method

**DOI:** 10.3390/nano11020280

**Published:** 2021-01-22

**Authors:** Tobias Esser, Tobias Wolf, Tim Schubert, Jan Benra, Stefan Forero, George Maistros, Stéphan Barbe, George V. Theodorakopoulos, Dionysios S. Karousos, Andreas A. Sapalidis, Evangelos P. Favvas

**Affiliations:** 1 Institute for Technical and Macromolecular Chemistry, University of Hamburg, Bundesstraße 45, 20146 Hamburg, Germany; tobias.esser@chemie.uni-hamburg.de; 2 Faculty of Applied Natural Sciences, TH Köln, Kaiser-Wilhelm-Allee, Gebäude E39, 51373 Leverkusen, Germany; tobias.wolf1@th-koeln.de (T.W.); stephan.barbe@th-koeln.de (S.B.); 3 Institute of Chemical Process Engineering and Plant Design, TH Köln, Betzdorfer Str. 2, 50679 Köln, Germany; tim.schubert@th-koeln.de; 4 Future Carbon GmbH, Ritter-von-Eitzenberger-Str. 24, 95448 Bayreuth, Germany; jan.benra@future-carbon.de (J.B.); stefan.forero@future-carbon.de (S.F.); 5 ADVISE, 17, Gymnasiarchou Madia St., 82100 Chios, Greece; gmaistros@advise-deta.com; 6 Institute of Nanoscience and Nanotechnology, National Center for Scientific Research “Demokritos”, Aghia Paraskevi, 15341 Athens, Greece; g.theodorakopoulos@inn.demokritos.gr (G.V.T.); d.karousos@inn.demokritos.gr (D.S.K.); a.sapalidis@inn.demokritos.gr (A.A.S.); 7 School of Chemical Engineering, National Technical University of Athens, 9 Iroon Polytechniou Street, Zografou, 15780 Athens, Greece

**Keywords:** cellulose acetate, mixed matrix membranes (MMMs), filler dispersion, ultrasonic sonotrode (USS), rotor-stator system (RS), impedance spectroscopy, CO_2_ separation

## Abstract

The main scope of this work is to develop nano-carbon-based mixed matrix cellulose acetate membranes (MMMs) for the potential use in both gas and liquid separation processes. For this purpose, a variety of mixed matrix membranes, consisting of cellulose acetate (CA) polymer and carbon nanotubes as additive material were prepared, characterized, and tested. Multi-walled carbon nanotubes (MWCNTs) were used as filler material and diacetone alcohol (DAA) as solvent. The first main objective towards highly efficient composite membranes was the proper preparation of agglomerate-free MWCNTs dispersions. Rotor-stator system (RS) and ultrasonic sonotrode (USS) were used to achieve the nanofillers’ dispersion. In addition, the first results of the application of the three-roll mill (TRM) technology in the filler dispersion achieved were promising. The filler material, MWCNTs, was characterized by scanning electron microscopy (SEM) and liquid nitrogen (LN_2_) adsorption-desorption isotherms at 77 K. The derivatives CA-based mixed matrix membranes were characterized by tensile strength and water contact angle measurements, impedance spectroscopy, gas permeability/selectivity measurements, and water permeability tests. The studied membranes provide remarkable water permeation properties, 12–109 L/m^2^/h/bar, and also good separation factors of carbon dioxide and helium separations. Specifically, a separation factor of 87 for 10% He/N_2_ feed concentration and a selectivity value of 55.4 for 10% CO_2_/CH_4_ feed concentration were achieved.

## 1. Introduction

Membrane systems possess many advantages such as small footprint, low capital and operating costs, being environmentally friendly, and no moving parts for separation and as such exhibit process flexibility [1]. However, there are several drawbacks of commercially available polymeric membranes (cellulose acetate (CA), cellulose triacetate (CTA), and polyimide (PI), etc.), especially when they are used for high pressure natural gas sweetening. In this regard, the relatively low separation performance (i.e., low CO_2_/CH_4_ selectivity) and low CO_2_ permeances, due to membrane compaction and plasticization, are the two major disadvantages. These issues lead to high costs due to a large required membrane area and a short lifetime, which indicates the need for developing novel, high performance membrane materials [2].

Among numerous types of membrane materials cellulose acetate is one of the oldest used and still remains a promising material with well-known properties for making defect free membranes. However, cellulose acetate, the “offspring” of cellulose nitrate, was made in membrane configuration for the first time by Brown [3] in 1910, and great attention is also given to it currently. The improved properties of CA, such as high biocompatibility, good desalting, high potential flux, good toughness, and relatively low cost classified the CA membranes in a high position [4,5]. Due to these characteristics, CA membranes have found wide uses in numerous applications, such as gas separations, microfiltration, and reverse osmosis-desalination [6]. Special attention is given to reverse osmosis applications thanks to the excellent hydrophilicity of the cellulose acetate, an important factor in the minimization of fouling phenomena [7,8]. 

Recently, mixed matrix membrane materials (“MMMs”) provided positive outcomes to membrane scientists, mainly thanks to their improved properties regarding selected gas selectivity, increased mechanical stability, lower plasticization, and inhibited degradation phenomena [9]. High attention is given to mixed matrix cellulose acetate-based membranes, structures which provide enhanced properties at both separation and anti-fouling [10,11,12,13,14]. From various nanomaterials proposed as appropriate fillers, nano-carbon materials (also referred to as “carbon nanomaterials”) are reported to yield new advanced properties on the final mixed matrix produced membranes. Carbon nanotubes (CNTs), both single and multi-walled (SWCNTs and MWCNTs), graphene oxide, and graphene nanoplatelet structures (GO, GNPs) are currently on the first line of membrane technology [15,16]. This new type of carbon-based mixed membranes in principle provides excellent properties for gaseous processes such as good CO_2_ permeabilities and selectivities versus CH_4_, H_2_, and N_2_ even under harsh conditions.

Carbon nanomaterials (CNTs, graphenes, GO, GNPs, fullerene, and various others such as nano-diamonds) are a fascinating class of materials owing to their special properties [17,18,19]. The interest of using nanoparticles as fillers in membrane structures focuses mainly on their beneficial effect on fluxes and fouling resistance [20]. Significant improvement in separation properties compared to neat polymers is expected for the resultant MMMs [21,22]. Particularly, the nano-carbon based materials added to a polymer matrix can induce the following three effects on the permeability properties: (i) they can act as molecular sieves altering the permeability, (ii) they can disrupt the polymeric structure increasing the permeability, and (iii) they can act as barriers reducing the permeability [23,24]. Moreover, nanocomposite materials render better thermal and mechanical properties.

In the present contribution, high purity carbon nanotubes, cellulose acetate, and diacetone alcohol were used to prepare flat sheet mixed matrix membranes, whereas the impact of the dispersion method (mainly ultrasonic and rotor-stator systems) on the final structural characteristics was also investigated.

## 2. Materials and Methods

**Figure 1:**
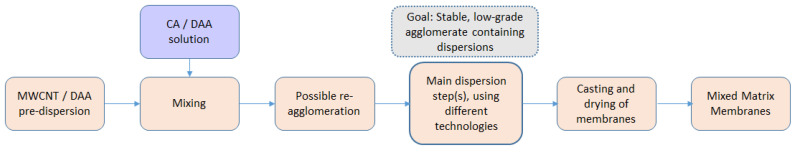
Schematic representation of the intermediate steps to be produced mixed matrix membranes (MMMs).

For preparation of the mixed matrix membranes both cellulose acetate (CA) and diacetone alcohol (DAA) were supplied from Sigma Aldrich (part of Merck Group, Frankfurter Strasse 250, Darmstadt, Germany). The multi-walled carbon nanotubes (MWCNTs) were obtained from FutureCarbon in pre-dispersion form, with a mass fraction of 1.5% or/and 2%, in DAA. Figure 1 describes the sequence of steps in the whole process, starting with the filler pre-dispersion and having as final target the achievement of mixed matrix membranes with improved gas separations. 

CA was dissolved in DAA under vigorous stirring, under the specific conditions which are described in Section 3. Subsequently, MWCNTs-dispersion (solution in DAA) was added once a homogeneous solution was obtained. Dispersion was finally achieved with a rotor-stator system, Silverson L5M (Silverson Machines, Inc. 355 Chestnut St., East Longmeadow, MA, USA). As comparison methods, an ultrasonification, by means of a sonotrode, and, for some additional experiments, a three-roll mill (TRM), were used after homogenization. The resulting solutions were cast with a wet thickness of 500 µm in a dedicated, dry casting device. It consists of a plate and a frame fixed by vacuum on the top of a tray. The plate acts as a substrate. In order to achieve the required wet thickness, a casting knife goes over the frame and removes excess material so that only solution remains within the frame. Thus, the height of the frame defines the wet thickness. To speed up the drying process, the plate is heated up to 60 °C. In this study, dry casting parameters were kept constant for all the prepared samples.

As an evaluation method of the dispersion quality, light (optical) microscopy was chosen. For this purpose, the dried membrane pieces were fixed on a microscope slide. A Motic BA310E microscope (C. Les Corts, 12 Pol.Ind, Barcelona, Spain) was employed with the Moticam 5+ and the software Motic Images Plus 3.0 ML. The agglomerate area was detected on the basis of a black and white balance. The software identified all black pixels and calculated the area based on a calibration. To reduce the error by comparing different samples, a relative agglomerate surface area was calculated as the ratio between the agglomerate surface area and the totally viewed surface area. This method is limited by the microscope resolution; only agglomerates can be detected whose particle size is greater than the resolution limit of the light microscopy (~1 µm). Small agglomerates, if they are wetted and in direct contact with the polymeric matrix material if they do not create, bear, pores or cause holes, might be tolerated, though an impact to permeation behavior is possible [10,25] and has to be investigated.

In addition, advanced characterization techniques such as tensile strength measurements on selected membrane samples, impedance spectroscopy, in order to evaluate the effect and the quality of the dispersion of MWCNTs, and gas permeability/selectivity investigation were carried out in order to evaluate the properties of the produced mixed matrix membranes.

## 3. Results and Discussion

**Figure 2:**
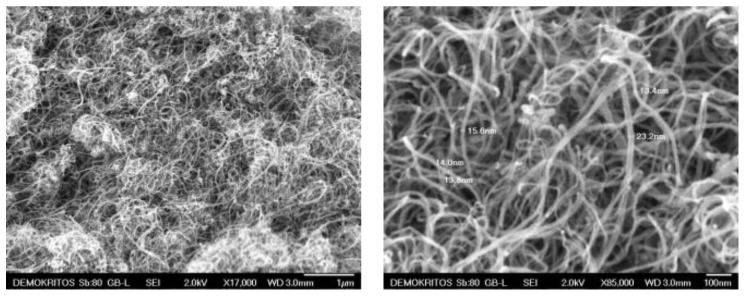
SEM images of multi-walled carbon nanotubes (MWCNTs).

High purity carbon nanotubes were produced by catalyst-assisted chemical vapor deposition [26,27]. In Figure 2, SEM micrographs illustrate the MWCNTs morphology and their interwoven and entangled arrangement. They appeared in the form of ribbon complexes with no sign of any impurities. The outer diameter of the MWCNTs ranged between 13 to 23 nm and their length fluctuated between 10–50 µm. 

**Figure 3:**
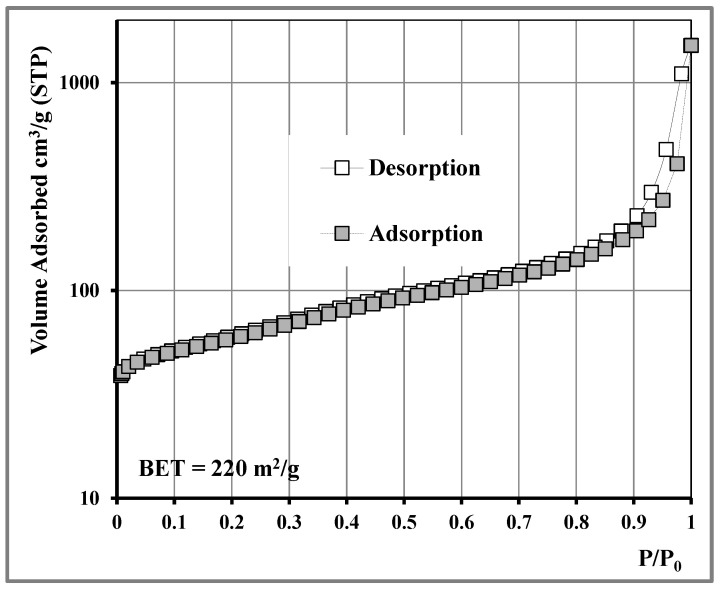
N_2_ adsorption isotherm at 77 K of MWCNTs.

Total pore volume, specific surface area, and pore size distribution characteristics were determined using liquid nitrogen adsorption measurements at 77 K. Prior to measurement, the samples were subjected to degassing overnight at 250 °C under high vacuum (10^−6^ mbar). The shape of the adsorption isotherm (Figure 3) was classified as type II with a close to type H3, mild adsorption hysteresis loop according to IUPAC classification [28]. The isotherm was similar to typical N_2_ isotherms and hysteresis for CNTs that are mesoporous materials with broad pore size distribution, according to the Kelvin equation, and plate-like particles aggregated into slit-shaped pore structures. The calculated specific surface area (SSA) was 220 m^2^/g according to the BET (Brunauer–Emmett–Teller) method. 

**Figure 4:**
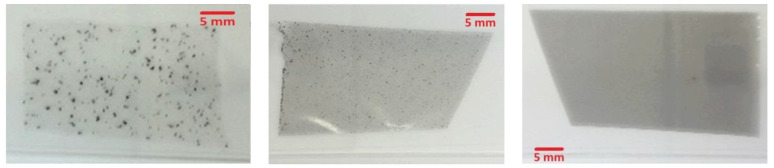
Dried films from preliminary study, without any secondary dispersion step. **Left**: 2.5 wt.% cellulose acetate (CA) solution (low viscosity), **middle**: 5 wt.% CA solution, **right**: 10 wt.% CA solution.

The aforementioned MWCNTs nanostructures were used as filler materials in order to prepare mixed matrix cellulose acetate flat sheet membranes. MWCNTs in DAA can be pre-dispersed using combined techniques with high shear rates leading to stable dispersions, but during long-term storage and especially when added to polymer solutions (which is necessary for membrane preparation), MWCNTs tend to re-agglomerate. After mixing (see also Figure 1, illustrating all intermediate steps), the parameter of the polymer concentration into the MWCNTs/polymer/solvent mixtures was determined as a long-term stability factor in the MWCNTs dispersions and an influencing factor for the drying process, as well as for the associated tendency of re-agglomeration during the membranes’ drying process. The dense CA and MWCNTs/CA mixed matrix membranes were prepared by applying the phase inversion method (precipitation by solvent evaporation). All the membranes were left for solvent evaporation up to 48 h in order to obtain dry film samples. Figure 4 depicts three dried films on object slides, prepared with mixing CNT-predispersion (2 wt.% MWCNTs) with polymer solution of different concentrations, 2.5, 5, and 10 wt.% CA and thus by different solution viscosities within a primary study. The strong impact of increased viscosity (by higher CA content), which leads to reduced sedimentation and mobility, is evident and therefore decelerates re-agglomeration tendency, but, in principle, cannot hold it from happening. Therefore, a secondary dispersion step becomes necessary (see Figure 1, showing behavior without secondary dispersion step), in order to avoid and withdraw re-agglomeration, thus distinguishing pre-dispersions (before mixing) and main (secondary) dispersion steps (after mixing), and to become less viscosity-dependent.

The secondary dispersion of the MWCNTs into the solvent was performed using both ultrasonic and rotor-stator techniques and the results were compared and discussed, recently supplemented with an additional dispersion using three-roll mill technology. For both main dispersion methods, the critical parameter is the energy input and transmission, which was systematically changed under the consideration of different treatment durations and equipment performances, which are described in Section 2. 

**Figure 5:**
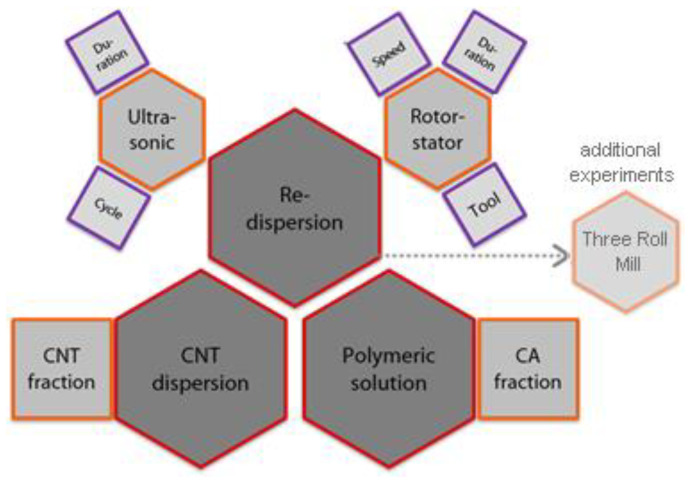
Summary of analyzed influencing factors in the parametric study of main dispersion step.

The impact of the dispersing method and the formulation of the polymer solution on the quality of the macroscopic structure and the properties of the produced membranes were thoroughly investigated. In Figure 5, the summary of influencing factors is presented as a sketch. To describe the influence of the individual parameters for each redispersing method, a parametric study is conducted according to the design of experiments (DoE). In the conducted parametric study, the parameters of each redispersing method varied between two levels labelled with (-) for the lower interval boundary and (+) for the upper interval boundary. 

Based on the aforementioned methodology, a large number of mixed matrix CA membranes were prepared. In Table 1, Table 2 and Table 3, the parameters for the used dispersion techniques are outlined and details of (**-**) and (**+**) settings are also described. 

According to DoE, based on parameters and values described in Table 1 and Table 2, 16 and 8 parameter combinations for the RS and USS methods, respectively, were required for determining the significance of each parameter. Furthermore, for the RS method a full factorial design of experiments was determined. This design covered a larger sample number (32 samples, each of them twice). Additionally, a second test plan was established, in which the step size between the parameter settings of rotor stator including treat duration and revolution speed was reduced. For the revolution speed, the settings ranged from 1000 min^−1^ to 8000 min^−1^ in steps of 1000 min^−1^. The duration of treatment was changed from 5 min to 30 min with 5 min step size. In Table 4, the main preparation parameters for the production of a series of CA mixed matrix membranes based on the RS and USS methods are described. 

**Figure 6:**
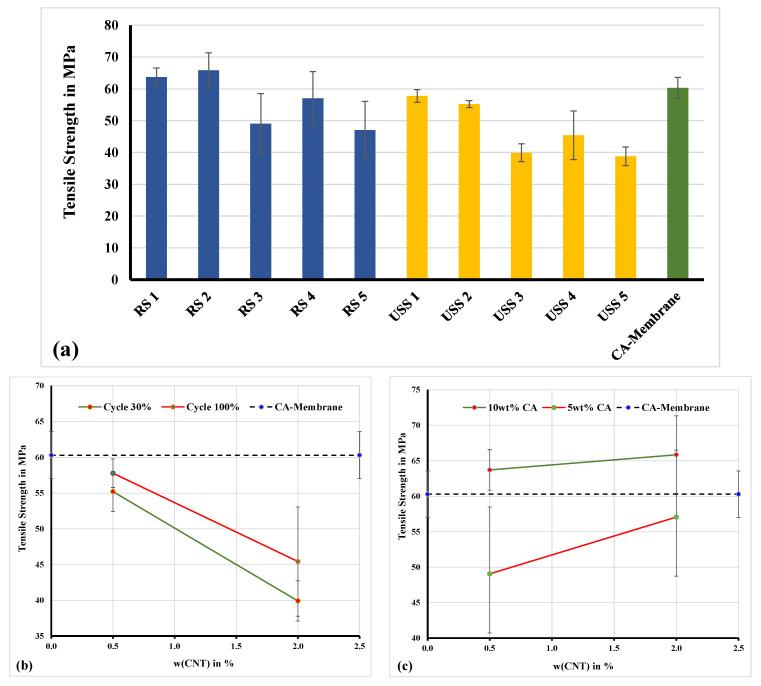
Mechanical analysis—(**a**) ultimate tensile strength of samples from Table 4. (**b**) Effect of MWCNTs concentration on tensile strength of membranes prepared by ultrasonification (USS). (**c**) Effect of MWCNTs concentration on tensile strength of membranes prepared by rotor-stator (RS).

The mechanical behavior concerning their ultimate tensile strength of selected membranes was also investigated [29]. In Figure 6a, a comparison diagram of the mechanical behavior between selected samples, which were prepared by using the ultrasonic sonotrode (USS) (Weber Ultrasonics AG Im Hinteracker, Karlsbad Germany) and rotor-stator system (RS) (Gydevang 4A, Allerød, Denmark), is presented. The ultrasonic sonotrode has a power rating of 200 W and was used to sonicate a solution volume of 160 mL.

All the measurements were performed at ambient humidity (~50%), whereas the samples were pre-equilibrated at this condition prior measurement. The measured tensile strength values are similar and even higher compared to similar membrane systems presented in the literature [10,30,31]. As it is shown in Figure 6a, the ultimate tensile strength was increased, compared to the CA-membrane sample, only in the cases of RS1 and RS2 samples. Both samples are prepared based on 10% of CA solution under conditions of 6000 rpm (Figure 6c). This can be addressed to the more localized heterogeneity, in the direction of increasing the tensile strength, developed by well dispersed MWCNTs particles. The well dispersed filler materials favor molecular interactions between the additive particles and the polymer and cause the macromolecules to form a tightly coiled conformation.

**Figure 7:**
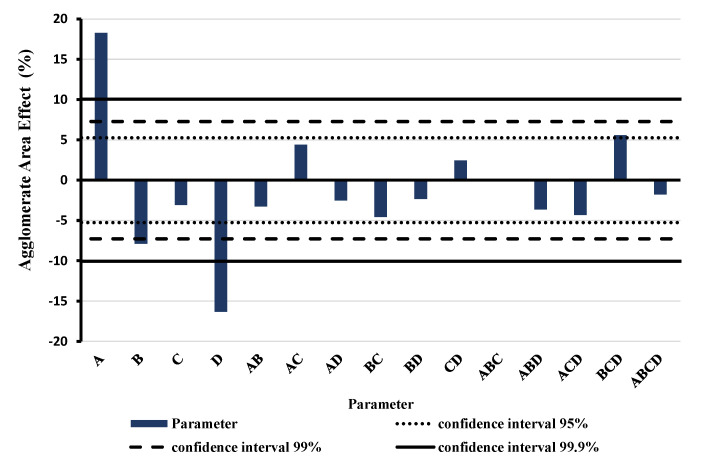
Results of the full factorial design of experiments of rotor-stator (RS) method. Blue bars show the effect of the single parameter (A = MWCNTs weight concentration, B = treatment duration, C = CA weight concentration, D = revolution speed) and the interactions between parameters based on the relative agglomerate area.

Based on the fact that the RS method can improve the tensile strength of the prepared membranes, a further study of the effect of each parameter was accomplished. Figure 7 shows the results of the DoE for full factorial design of RS method depicting the effect of the parameter variation and the interaction between them. A (purely numeric) positive effect in the diagram means an increasing of the relative agglomerate area and consequently a less effective dispersion. The highest and most significant effect is shown by the weight concentration of the MWCNTs (A)—here a high concentration worsens dispersion and the revolution speed (D)—here a higher speed improves dispersion.

It has to be mentioned that variation of parameters was achieved by increasing quantity of MWCNTs dispersion in DAA leading to solvent concentration increment during preparation. Increased DAA concentration can have several, partially opposite effects. High concentration and lower viscosity enhance CNT mobility (which can increase re-agglomeration rate). However, in case of RS and USS dispersion, a high DAA concentration also facilitates dispersion progress. On the other hand, especially in the case of the three-roll mill dispersion technique, a higher viscosity improves shear stress transfer in the gap between the rolls. Overall, this system is multifactorial and that is why the design of experiments is a crucial factor that must be analyzed by taking into consideration all the involved parameters and the interaction between them. 

In addition to these two dominant effects, there are two other significant parameters; one of them is the treatment duration (B) and the other is the interaction between treatment duration, revolution speed, and CA weight concentration. Optimal, low agglomerate area was found for low MWCNTs weight concentration (0.5 wt.%), long treatment duration (30 min), high CA weight concentration (10 wt.%—though not significant) and high revolution speed (6000 rpm).

**Figure 8:**
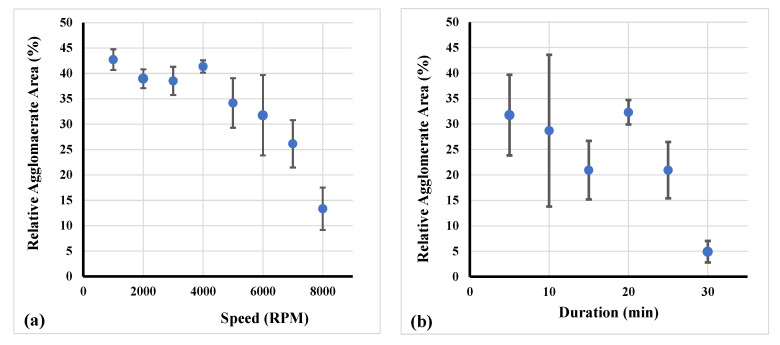
Relation of the relative agglomerate area by changing (**a**) revolution speed and (**b**) treatment duration of RS.

As described in Section 2, a second series of experiments (every experiment was repeated three times) was conducted and respective results are presented in Figure 8. Specifically, the correlation of agglomerate area and revolution speed is presented in Figure 8a. A higher speed led to a decline of the relative agglomerate area corresponding to a convex curve type. Furthermore, in Figure 8b, the relation of the relative agglomerate area with the treatment duration increment is shown. As displayed, the agglomerate area decreased with increasing duration of treatment. After treatment duration of 25 min this tendency increased with an even steeper decline.

Summarizing the results, arranged according to dispersion technology, it is obvious that:

(I) In the USS system: (1) any increase of MWCNTs pre-dispersion concentration leads to weakening of the material due to more poorly dispersed MWCNTs in secondary dispersions; (2) the decrease of run cycles results in poorly dispersing conditions and yields more agglomerates; (3) this method generates more inhomogeneous zones within the polymer matrix, which causes the material to be weaker; and (4) it is the less suitable method.

Due to the correlation of mechanical tests results and microscopic images of the membranes, these remarkable observations can be confirmed. It could be clearly found that the number and size of the agglomerates increased significantly with increasing MWCNTs concentration. This complies with the assumption that the increase in MWCNTs concentration leads to weakening of the material by more poorly dispersed MWCNTs. It is plausible that the increased number of particles leads to an increased attenuation of the ultrasonic waves and thus significantly reduces the energy transmission.

(II) In the RS system: (1) the increase of the MWCNTs pre-dispersion concentration leads to a reinforcement of the material by well-dispersed MWCNTs; (2) the decrease of CA concentration results in thinner, less stable membranes; (3) a weak interaction is recorded between CNTs wt.% and CA wt.%; (4) the smaller the CA concentration, the higher the effect of the MWCNTs concentration; and (5) finally RS seems to be the more suitable method for the MWCNTs dispersion.

Excellent dispersion stability was found using LuMiSizer^®^ equipment (LUM GmbH, Justus-von-Liebig-Str. 3, Berlin, Germany), which combines extinction of suspensions with centrifugation-enhanced sedimentation. The measurement shows light transmission as a function of position and time—low transmission and the lack of a clear supernatant even after total duration of centrifugation (relevant position is the filled area between 112.5 and 124.6 mm) proves and quantifies stability of dispersions. The measuring parameters for LuMiSizer stability tests were: T = 25 °C, 900 profiles all 10 s (ca. 2.5 h total duration), 3000 rpm (centrifugal force: 1308 g and rotor length 13 cm), and high light factor due to strong light absorption. No sedimentation was detected for any of the 6 representative samples (RS dispersions). 

**Figure 9:**
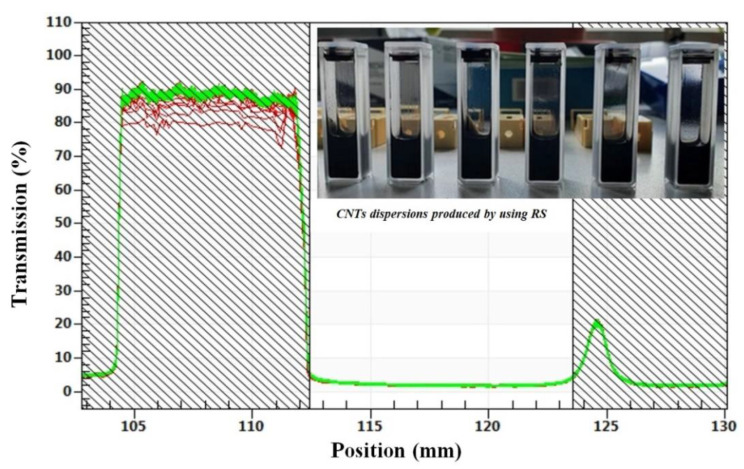
Transmission profiles for representative RS method CA/MWCNTs samples after centrifugation for 2.5 h in LuMiSizer equipment exhibiting no sedimentation or instability.

**Figure 10:**
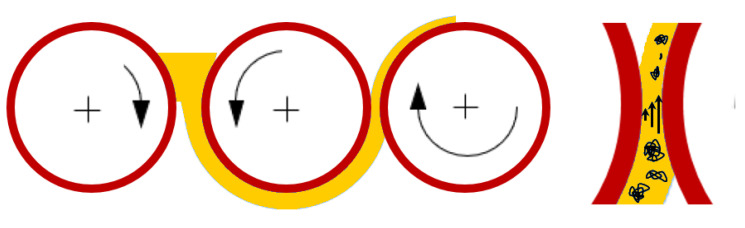
Principle of three-roll mill (TRM) method and shear force transmission within the fluid in the defined gap is decrease agglomerate size.

In Figure 9, the excellent stability of 6 representative samples, using RS dispersion technology, can be seen; transmission of light during extinction analysis did not increase during 2.5 h of centrifugation and dispersion instability index ranges between 0.004 and 0.007 for all samples. At this point, it must be noted that values below 0.01 indicate stable dispersions in long term. However, comparing the dispersion methods I (RS) and II (USS), USS cannot disentangle and minimize agglomerate size, but improves CNT–CA matrix interaction, a sort of close-up effect. RS is more suitable for minimizing agglomerate sizes, but does not provide a close-up effect and that is why the final recorded gas selectivity performance is improved in the case of the mixed matrix membranes prepared by the USS method. 

(III) Promising first results using three-roll mill (TRM) technology (see Figure 10) were deduced; additional dispersion experiments have been added using the TRM method. The motivation is that for several other nanocarbon dispersions, TRM achieved good agglomerate size reductions and quite homogenous size distributions. A three-roll mill of the type Exakt 80E plus (EXAKT Advanced Technologies GmbH, Norderstedt, Germany) was used with the following machine parameters: final gap width (second to third roll) 6 µm and 580 rpm rotational speed.

In TRM technology, shear forces are caused by the different rotational speeds of the rolls, in the gap and are transmitted solely by the fluid. Therefore, for the TRM method, higher viscosities (in contrast to many other dispersing methods) are helpful to reduce agglomerate size. 

**Figure 11:**
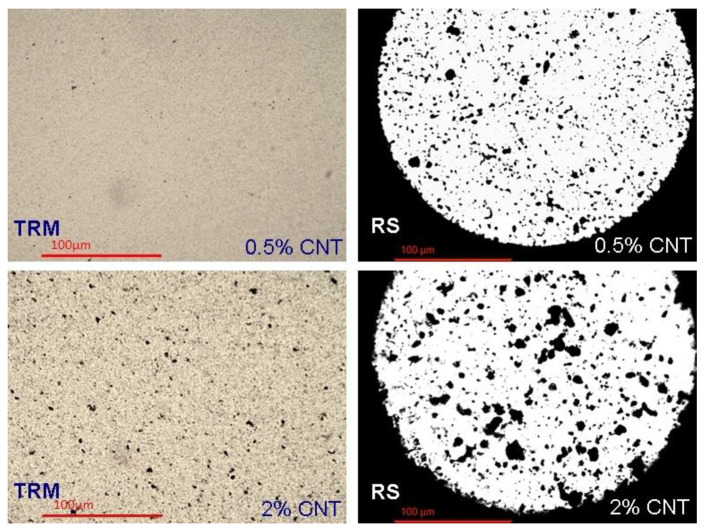
Variation of dispersion method and MWCNTs content: **Left** side: TRM method, **right**: RS method, above: 0.5 wt.% MWCNTs, below: 2 wt.% MWCNTs.

Figure 11 illustrates considerably improved dispersion and reduced agglomerate size, even compared to RS method for both (0.5 and 2 wt.%) MWCNTs’ concentrations. 

**Figure 12:**
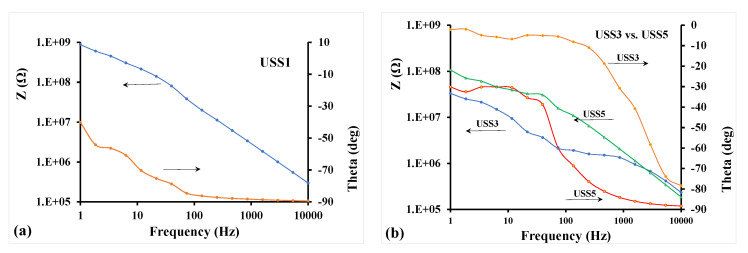
(**a**) Impedance spectrum of membrane sample USS1; (**b**) Effect of higher concentration of MWCNTs and of cycle duty (30 and 100%), on the impedance spectra of samples USS3 and USS5.

In addition, the mixed matrix cellulose acetate flat sheet membranes were examined by impedance spectroscopy in order to evaluate the effect and the quality of the dispersion of MWCNTs. For these tests, the membrane samples were positioned on flat interdigital dielectric sensors thus facilitating single-side electric field application on the samples. The membranes were pressed on the sensor surface and a bias voltage of 10 V AC at frequencies from 1 Hz to 10 kHz was applied at the sensor terminals [32,33]. The impedance vector was estimated and was expressed in the form of impedance amplitude, Z, and phase angle, theta (θ). A typical result is depicted in Figure 12a for the USS1 membrane sample, which contains 0.5% MWCNTs. The spectra show high Z values at the low frequency range and θ values raised above −90° only at low frequencies. This electrical behavior of the membrane is capacitive indicating relatively large mean distance between MWCNTs particles in the dispersion. Given the low concentration of MWCNTs and full cycle duty in dispersion, this result is well expected [34]. It is believed that this type of measurement and assessment of membrane electrical behavior can be used for the quality check of the membrane condition during operation in gas cleaning.

Figure 12b demonstrates the sensitivity of the impedance spectra of the membranes on the MWCNTs content and the dispersion time. Here the concentration of MWCNTs was increased to 2% for both USS3 and USS5 membranes included in Figure 12b. Lower values of Z at low frequencies were observed for both membranes as compared to USS1 sample of Figure 12a. The effect of cycle duty in dispersion was also represented at the impedance spectra: USS3 sample subjected to lower cycle duty (30%) exhibits much lower Z values at low frequencies, meaning higher conductivity, than USS5 sample, which had full cycle duty (100%). This feature is indicative of lower mean distance between MWCNTs particles for USS3 compared to USS5. Furthermore, USS3 sample exhibited values of θ close to 0° across the low frequency range, thus a resistive behavior. When the cycle duty of dispersion increased to 100% for USS5, θ values drop across the range to restore a capacitive behavior of the membrane, which is characteristic of large mean distance of nanoparticles (more effective dispersion). 

The impedance vector will be quantitatively analyzed in the next steps by equivalent circuit models, i.e., branches of capacitors, resistors, and inductances, where each circuit element corresponds to a physical parameter, such as size of MWCNTs agglomerates and average distance between MWCNTs particles in the polymer matrix. While the quality of dispersion will be assessed through the equivalent circuit modeling of the spectra, it can be observed that the membranes are below the conduction percolation threshold and their resistive behavior can be used as a quantitative measure of the above contributions. 

Additionally, the gas permeability and selectivity study of selected mixed matrix CA membranes indicates the important impact of the different preparation methods. The capability of a membrane to separate gas mixtures is reported in literature mainly in terms of two separation factors: (1) the ideal selectivity factor, and (2) the mixture selectivity factor. The ideal selectivity $a_{ij}=\frac{P_{i}}{P_{j}}$, is defined as the ratio of the permeabilities *P_i_* and *P_j_* of two pure gases, measured separately under the same conditions, with *i* being the most permeable gas. The real selectivity is the result of the online analysis of the membranes permeate gas streams, *i* and *j*, as monitored by using a gas chromatography analysis or/and gas analyzers. In any case, what really matters is the membrane’s selectivity property of a real binary mixture feed and of multicomponent mixtures which are closer to what exists in the industrial processes.

A high number of different gases need to be separated in numerous industrial processes. Among them, the separation of helium and carbon dioxide are stated in the high priority of the natural gas industry, mainly, for energy and environmental interest. Helium, one of the gases which can escape to the universe, is produced primarily from natural gas (NG). It is encapsulated in NG reservoirs over many hundreds of years as the result of radioactive decay of uranium and thorium in the Earth’s interior [35]. The largest reserves of helium-rich natural gas fields exist in western USA, New Mexico and Alaska, USA, but also in Qatar, Australia, Poland, and Algeria. Potential fields are reported to be in Russia, Iran, Italy, Tanzania, and India [36,37]. Since the commercial uses of helium increase and the problem of gas loss out of the Earth’s atmosphere arises, novel technologies for helium recycling and recovery from NG sources are sought after. As the separation of helium from natural gas has been suggested as a competitor to the cryogenic method currently employed, the study of both inorganic and polymeric membranes is of high interest in the membrane community.

Gas transport through dense polymeric membranes could be described reliably by the solution–diffusion model. According to the solution–diffusion model, main steps of the gas permeation mechanism are three: (1) first the gas molecules dissolve into the polymer matrix, among the chains; (2) afterwards the molecules move to the free volume areas according to the concentration gradient; and (3) finally the gas is desorbed, or evaporated, on the other side of the membrane. The parameters that define the permeability properties in the case of a dense polymeric membrane are their matrix properties such as density, rubbery or glassy nature, existence of free volume, etc. Another critical reason for the gas permeability differences, among different polymers, is the variations of the physicochemical interactions between gas molecules and the polymer chains for the various types of polymers. In these systems, the permeability results as the product of the solubility coefficient and the diffusion coefficient according to the equation P=D·S, where *P* is the permeability coefficient, *D* is the diffusion coefficient, and *S* is the solubility coefficient [38]. Given the fact that helium is an inert gas, the only interaction between the gas and the polymer matrix is based on the dispersion forces, the dual-sorption model cannot be applied. The Henry’s Law constant of helium in any polymeric membrane will merely be a function of the volume fraction of the polymer [37,39].

In the mixed matrix polymeric membranes, the polymer–particle interface morphology is a critical parameter to determine the overall gas transport properties. Three factors contribute to the poor interface characteristics including: (1) low adhesion between polymer and particle; (2) partial blockage of the particle pores by polymer chains; and (3) polymer chain rigidification. Low adhesion between materials could lead to the formation of non-selective voids at the interface region. Consequently, these voids act as extra channels to allow gas molecules to transport through, thus increasing the permeability and at the same time reducing the selectivity of the whole membrane [40].

**Figure 13:**
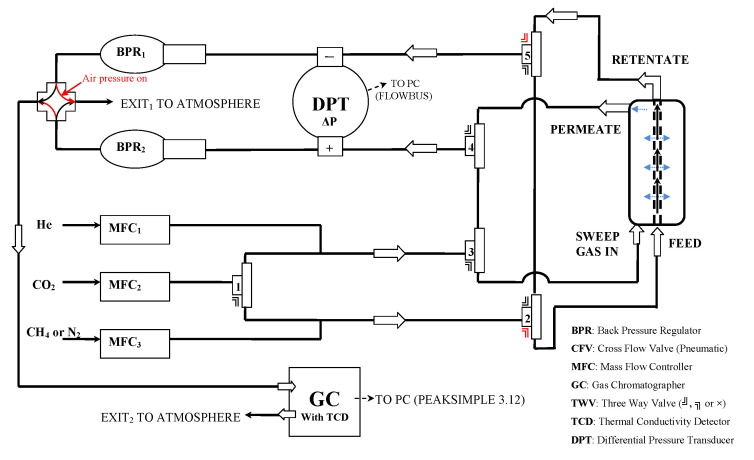
Scheme of membrane testing rig for gas separation measurements under continuous flow.

Gas separation studies under continuous flow were performed with the rig of Figure 13 [41]. The membrane module was connected to the four terminals of the rig and kept at the desired temperature by a heating tape element, controlled by a YUMO d-Tron 316 PID (Mackenrodtstraße 14, Fulda, Germany) temperature controller, while the pressure drop across the membrane was regulated by two Bronkhorst (Bronkhorst High-Tech B.V., Nijverheidsstraat 1A, AK Ruurlo, Netherlands) back pressure regulators BPR1 and 2 at the retentate (head-pressure) and the permeate side, respectively. The membrane feed stream concentration was regulated through proper adjustment of flow rates by Bronkhorst mass flow controllers (MFC2 for CO_2_ and MFC3 for CH_4_ or N_2_), while keeping the total feed stream at 100 ccSTP/min. Helium or argon was used as the sweep gas for sweeping the permeate side of the membrane, with a flow rate of 30 cc/min, controlled by mass flow controller MFC1 in Figure 13. All flow rates were measured with a Supelco Optiflow 520 bubble flow meter (Sigma-Aldrich, part of Merck Group, Frankfurter Strasse 250, Darmstadt, Germany) at the exit to atmosphere. Gas concentrations were measured with an SRI 8610C gas chromatograph (SRI Instruments20720 Earl St. Torrance, CA, USA) equipped with a fused silica capillary column and a TCD detector.

Selectivities (i.e., gas separation factors) were calculated from the following equation: $S=\frac{{\left(y_{gas1}/y_{gas2}\right)}_{permeate}}{{\left(y_{gas1}/y_{gas2}\right)}_{feed}}$, where *y_i_* are the gas concentrations expressed as % *v*/*v*. Permeability of each gas was calculated from the equation: $Pe_{i}=10^{-10}\cdot \frac{y_{i}\cdot Q\cdot dx}{A\cdot dP_{i}}$, where $Pe_{i}$ is the permeability of gas *i* in Barrer (where 1 Barrer = 10^−10^ cm^3^·(STP)·cm·cm^−2^·s^−1^·cm·Hg^−1^), *Q* is the volumetric flow rate at the permeate exit (cm^3^·(STP)·s^−1^), *y*_i_ the concentration of gas *i* in the permeate stream (net number, % *v*/*v* concentration of feed gas stream), *A* the effective area of the membrane (in cm^2^), d*x* the thickness of the membrane’s separation layer (in cm), and d*P*_i_ the partial pressure drop across the membrane for gas *i* (in cmHg). In all gas permeation tests, the surface area of the membrane was 5.3 × 10^−4^ m^2^ and the membrane thickness was about 20 μm.

Two separations were selected to be studied in our work, He/N_2_ and CO_2_/CH_4_ separations. Both separations are processes which belong to natural gas industry, the first one in the helium production/separation process from natural gas streams and the second in the natural gas sweetening process.

In the case of He/N_2_ separation, 10% He in N_2_ gas mixtures were fed to one side of the membrane under continuous flow conditions, while argon gas was sweeping the other side of the membrane. For CO_2_/CH_4_ separation experiments, 10% CO_2_ in CH_4_ gas mixtures were fed to one side of the membrane under continuous flow conditions, while helium gas was sweeping the other side of the membrane.

**Figure 14:**
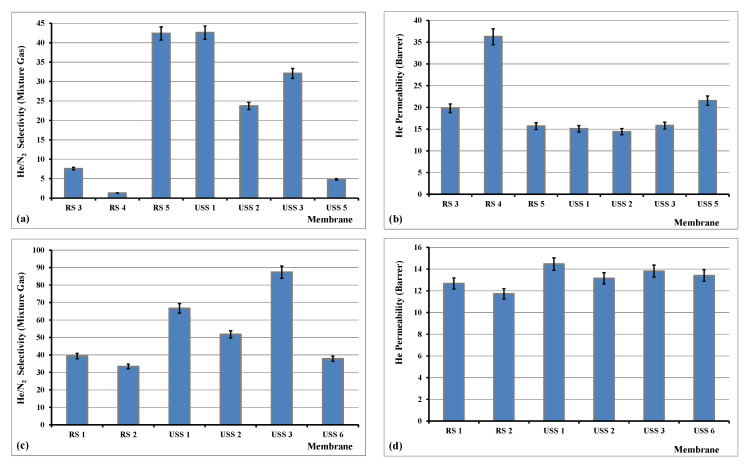
Selectivity of He/N_2_ gas mixture and permeability of helium at dP of 1.33 bar and 28 °C (**a**,**b**), and selectivity of He/N_2_ gas mixture and permeability of helium at dP of 5 bar and 28 °C (**c**,**d**).

**Figure 15:**
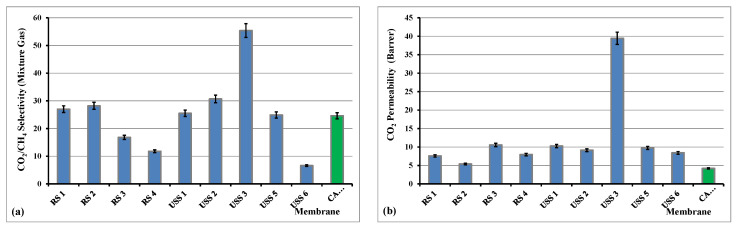
Selectivity of CO_2_/CH_4_ gas mixture (**a**) and permeability of CO_2_, (**b**) at dP of 1.33 bar and 28 °C.

He/N_2_ separation experiments took place under dP pressure (between feed and permeate streams) under conditions of 1.33 and 5 bar(a), whereas for CO_2_/CH_4_ no significant effect was performed at higher dP conditions and only the measurements of the experiments at dP of 1.33 bar(a) are presented and discussed. It must also be noted that for pure CA membrane, in the case of He/N_2_ selectivity experiments, the helium permeability measurement was achieved only in the feed pressure of 7 bar, where the helium permeability was calculated equal to 10 with a He/N_2_ selectivity of 37.2. At lower feed pressures, 1.33 and 5 bar, helium was not detectable with the high sensitivity TCD detector. All the prepared membranes were tested in both 10% He/N_2_ and 10% CO_2_/CH_4_ mixtures and the permeability/selectivity properties presented in Figure 14 and Figure 15 correspond only to the samples which are performing high selectivities in aid of helium and CO_2_. Here it must be noted that all the presented values are referred to gas mixture selectivities and not to ideal calculated properties.

From the decade of the 1970s, among others, cellulose acetate membranes were mentioned as candidate material for this application [37]. Although numerous polymeric membrane materials have been evaluated on various gas mixtures only few of them have been investigated with He/N_2_ separation. Specifically, in the work of Gatzel and Merten [42], an ideal separation factor of 97 was measured for a dense cellulose acetate membrane. However, the helium permeability was measured equal to 1.36·10^−9^ cm^3^(STP)·cm/cm^2^·s·cm(Hg). Polyvinylthrimethilsilane (PVTMS) membranes were, also, tested on He/N_2_ separations before and after a gas fluorination process/treatment. The selectivity increased from 9 to 35 after the fluorination treatment [43]. PDMS poly(imide siloxane) and the derivative CNTs composite membranes have also been tested on He/N_2_ separations. The achieved separation factors (ideal) were very low, from 3.3 to 4.6 [44]. On the other hand, inorganic membranes, mainly carbon molecular sieve membranes, provided very high He/N_2_ selectivity properties. For example, carbon molecular sieve membranes prepared from P84 polyimide precursors provided He/N_2_ selectivities higher than 230 [45,46,47,48]. In addition, carbon molecular sieve (CMS) membranes were prepared on mesoporous γ-alumina support by pyrolysis of defect free 6FDA-based polyimide polymer film. Mixture He/N_2_ selectivity was measured equal to 55 in a membrane with a thickness of about 300 nm [49]. 

In our case and as it is shown in Figure 14, the achieved He/N_2_ selectivity fluctuated between 1.3 and 87.4. In specific, the highest measured selectivity was 87.4 for the sample USS3, which was prepared under USS method and was loaded with 2% of MWCNTs. This value of 87.4 was achieved in dP of 5 bar, much higher than 32.1, which was measured for dP of 1.33 bar. Further increase of dP did not improve the He/N_2_ selectivity properties of this membrane. The helium permeability was measured between 12 and 37 Barrer. These recorded values are equal or/and higher than what is reported in the literature [50]. 

Another crucial industrial separation is that of CO_2_/CH_4_ as, among other separation processes, it is one of the basic separations during the natural gas sweetening. The large amounts of CO_2_ which are emitted to the atmosphere are associated with fossil fuel consumption to fulfill the energy demands for power generation, transportation, industry needs, and other anthropogenic activities, which are generally accepted as the leading cause of climate change and global warming [51]. Until today, the current CO_2_ capturing at large scale takes place mainly via regenerative absorption of CO_2_ using alkanol amines [52]. On the other hand, new operations use membrane technology for the removal of CO_2_ and other impurities from natural gas due to the space and weight limitations of the off-shore sites [53,54].

Numerous polymeric materials, both glassy and rubbery, have been tested so far for CO_2_ separation applications. Well-known glassy polymeric materials for CO_2_ separations are cellulose acetate (CA), polyimide (PI), and polysulfone (PSF) (Baker, 2002; Baker, 2009; Bernardo et al., 2009) [55,56]. Rubbery polymers such as PDMS, polyvinyl alcohol (PVA), and Pebax^®^ are capable of being more selective towards heavier gases such as CO_2_ over smaller molecules, e.g., He or H_2_ [57,58,59]. The glassy polymers are characterized by low free volume. This is the main reason that these membranes demonstrate low gas permeability but significantly high gas selectivity, even at high operating pressures. On the other hand, in rubbery polymer membranes, the selectivity is based on the sorption phenomena and usually low diffusion selectivity is obtained. Especially, in the case where the rubbery polymers have low glass transition temperature (T_g_), equal or/and below room temperature, the chemical affinity between gas molecules and polymer chains is enhanced, mainly, due to the flexible chain motion in the rubbery state [60].

Among other glassy polymers, cellulose acetate is classified as one of the current industrial standards for the removal of CO_2_ from natural gas [61]. CA is a polymer with good selectivity properties of both CO_2_/CH_4_ and H_2_S/CH_4_ separations, as it has been evaluated in triune feed composition of H_2_S/CO_2_/CH_4_ gases [61,62].

The behavior of the observed increased CO_2_ permeability and decreased CO_2_/CH_4_ selectivity with increasing feed pressure has recently also been reported for cellulose acetate hollow fiber membranes [63]. This occurred for the sample USS7, as it is presented in Table 4. The observed CO_2_/CH_4_ selectivity values are similar, even better, to what is reported in the literature for this kind of material [61,64,65,66,67].

The effect of the higher feed pressure did not change remarkably the separation coefficient of the CO_2_/CH_4_ and for that reason the relevant data of all the studied samples were not presented. On the contrary, the sample USS7 was not permeable for CO_2_ at the pressure of 1.33 bar and the CO_2_/CH_4_ selectivity could only be measured above 5 bar. Further increase of the feed pressure did not affect the CO_2_ permeability and CO_2_/CH_4_ selectivity properties. It is obvious that the sample USS3, with 2% concentration of MWCNTs, provided the highest selectivity coefficient for CO_2_/CH_4_ separation and also one of the higher selectivity values for He/N_2_ separation. As it is shown in Figure 15, the CO_2_ permeability increases for all the studied membranes, especially significantly in the case of USS3 membrane, compared to CA pure sample, whereas the CO_2_/CH_4_ mixture selectivity is recorder lower in the cases of RS3, RS4, and USS6 membranes, higher for RS1, RS2, USS1, USS2, and USS5, and significantly higher for USS3 membrane.

In both studied separations, He/N_2_ and CO_2_/CH_4_, the main separation mechanism is that of solution-diffusion. However, by adding the nanofillers into the cellulose acetate matrix, new diffusion paths are created and these “porous-like channels” contribute to the gas permeation performance. The existence of the interface spaces, between filler and polymer chains’ surfaces, decreases the total resistance of gas flux and that is why the increase of the helium and CO_2_ permeability is observed for all the mixed matrix derivative membranes compared to the pure cellulose acetate. Furthermore, in the case of CO_2_/CH_4_ mixture the higher adsorption coefficient of CO_2_ into filler material could explain the increase in the selectivity values for some mixed matrix membranes, like USS2 and especially for USS3.

Additionally, a huge global issue is the existence of clean drinking water. Unfortunately, the available drinking water reserves are diminishing [68]. Aiming to the recovery of this phenomenon, the development of new nanofiltration (NF), reverse osmosis (RO), forward osmosis (FO), membrane distillation (MD), and membrane crystallization (MC) materials and processes are currently an appropriate choice for brackish and seawater treatment. Thus, the development of materials assuring high fluxes and salt rejection and functioning at lower pressures is of great interest. Based on this issue, already known and well-established membrane materials were again tested on water permeability and salt rejection evaluation.

**Figure 16:**
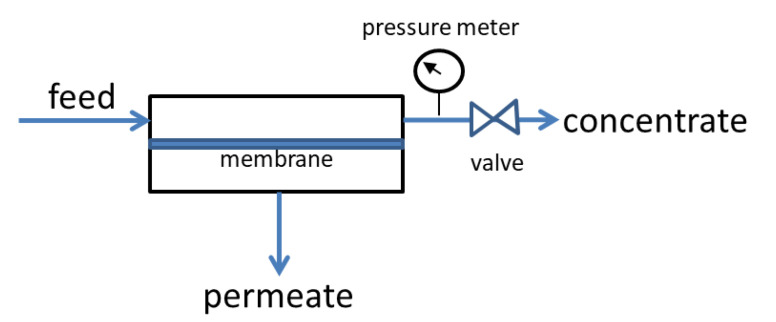
Simplified scheme of the water permeation setup.

To this end, the aforementioned CA based membranes were also tested on water flux measurements and the effect of the filler materials was recorded with high importance. The membranes were placed in a 5 × 5 cm (25 cm^2^) active area cross flow cell as depicted in Figure 16. Water at 20 °C was fed into the membrane cell with a flowrate of 100 mL/min with the aid of an electronically driven gear pump, while the pressure was adjusted to the desired value of 5 bar with the aid of a manual metering valve. Feed and permeate flow were recorded constantly with the aid of two inline Bronkhorst Cori-Flow™ (Bronkhorst High-Tech B.V., Nijverheidsstraat 1A, NL-7261 AK Ruurlo, Netherlands) Coriolis mass flow meters.

Mass transport though a non-porous membrane is generally accepted to be expressed by the solution-diffusion model. According to that, permeation takes place following a series of steps. In simple terms, first the dilution of molecules in contact with the membrane’s surface takes place which then they disuse through the volume of the membrane, it reaches its external surface and then desorbs at the permeate side. Several theories have been proposed in order to approach the overall diffusion of species though a polymeric dense membrane with Maxwell-Stefan being the most generally applied. In respect to desalination the following relationship gives the solvent’s flux [69]:

$$J_{w}=\frac{P_{w}\;}{\Delta \chi }\left(\Delta P-\Delta \Pi \right)=A_{\begin{array}{c} w \end{array}}\left(\Delta Ρ-\Delta \Pi \right),\;A_{w}\;=\;P_{w}/\Delta \chi$$

where *P_w_* is the solvent permeability, Δ*χ* is the membrane’s thickness, Δ*P* is the pressure difference and Δ*Π* the osmotic pressure difference across the membrane, and *A_w_* is the solvent permeability constant. 

Solute rejection is given by:

$$R={\left[1+\left(\frac{\rho_{w\;}A_{s}}{A_{w}}\right)\left(\frac{1}{\Delta P-\Delta \Pi }\right)\right]}^{-1}$$

where *R* is the solute rejection and *ρ_w_* is the solvent’s density.

The mixed matrix membranes are characterized by an open path network with size much larger than typical hydrated ions, and this kind of membrane is not appropriate for NaCl rejection. On the other hand, preliminary results show that the studied MWCNTs/CA membranes could be good candidates for other separations such as dyes removal from paint factory wastewater. 

Table 5 describes the pure water flux characteristics, in L/h/m^2^, of the selected studied membranes at a feed pressure of 5 bar.

As shown in the table, the addition of the filler material into the cellulose acetate matrix decreases remarkably the water flux. This can be attributed to the increased path, which was formed by the addition and the good dispersion of the MWCNTs, through which the water molecules pass the membrane [70]. This open path network, combined with the fact that the filler material provides an extra barrier to the water permeability, increases the water permeation resistance and finally leads to a decreased flux through the membrane.

## 4. Conclusions

By using MWCNTs as filler, a large number of mixed matrix cellulose acetate flat sheet membranes were prepared and characterized. Ultrasonic sonotrode (USS) and rotor-stator system (RS) were used for the dispersion of nanostructured carbon materials into DAA solvent. The parametric study of the effect of numerous parameters of both USS and RS techniques took place by concluding that the RS method seems to be the more suitable method for the MWCNTs dispersion. Most recent results also show that the TRM dispersion method offers an opportunity for further improvement of dispersion. Mechanical properties investigation of the prepared membranes was accomplished by determining the ultimate tensile strength. Due to the correlation of results of mechanical tests and microscopic images of the membranes, it was concluded, as expected, that the number and the size of the agglomerates increases significantly as the MWCNTs concentration increases. In addition, the significant parameters for the RS system could be identified. A closer look at the revolution speed and treatment duration enabled us to describe the relation between these parameters and the relative agglomerate area. Furthermore, the prepared membranes were tested by impedance spectroscopy in order to evaluate the effect and the quality of the dispersion of MWCNTs. The obtained results indicate that increased polymer concentration, increased concentration of MWCNTs, and increased time of dispersion lead to higher conductivity values of the prepared membranes. Finally, the membranes present good properties for both CO_2_/CH_4_ and He/N_2_ separations with the highest achieved mixed gas separation coefficients of 55.4 and 87, respectively. The addition of the filler material into CA matrices also improves also the water barrier properties of the mixed matrix membranes.

## Figures and Tables

**Table 1:** Parameters of rotor-stator (RS) system.

Parameter	-	+
Treatment duration (min)	5	30
Revolution speed (min^−1^)	2000	6000
Tool (geometry stator)	Emulsor screen (ES)	General purpose disintegrating head (MUSS)
MWCNTs weight concentration (%) *	0.5	2
CA weight concentration (%)	5	10

* referred to dried membrane.

**Table 2:** Parameters of ultrasonic sonotrode (USS).

Parameter	-	+
Treatment duration (min)	2.5	5
Cycle (%)	30	100
MWCNTs weight concentration (%) *	0.5	2
CA weight concentration (%)	5	10

* referred to dried membrane.

**Table 3:** Parameters of three-roll mill (TRM).

Parameter	Value
Roll gap (µm)	10
Revolution speed (min^−1^)	450
Roll temperature (°C)	24
MWCNTs weight concentration (%) *	0.5–2
CA weight concentration (%)	10

* referred to dried membrane.

**Table 4:** Parameters used for preparation of samples for mechanical analysis.

**Samples Prepared by Rotor-Stator System**					
**Sample**	**Method**	**CA Concentration (%)**	**MWCNTs Concentration (%)**	**Revolution Speed (min^−1^)**	**Duration (min)**
RS1	RS (ES)	10	0.5	6000	30
RS2	RS (MUSS)	10	2.0	6000	30
RS3	RS (MUSS)	5	0.5	6000	30
RS4	RS (ES)	5	2.0	6000	30
RS5	RS (MUSS)	5	0.5	2000	5
**Samples Prepared by Ultrasonification**					
**Sample**	**Method**	**CA Concentration (%)**	**MWCNTs Concentration (%)**	**Cycle (%)**	**Duration (min)**
USS1	USS	5.0	0.5	100	2.5
USS2	USS	10.0	0.5	30	2.5
USS3	USS	10.0	2.0	30	5
USS4	USS	10.0	2.0	100	2.5
USS5	USS	5.0	2.0	100	5
USS6	USS	10.0	0.5	100	5
USS7	USS	10.0	2.0	100	2.5

**Table 5:** Water permeability properties, in L/(h·m^2^·bar), for seven selected CA based membranes.

Sample	Water Permeability (L/(h·m^2^·bar))	Water Permeability Decrease (%)
CA-Membrane	109	--
RS1	36	67
RS2	38	65
USS1	23	79
USS2	27	75
USS3	12	89
USS6	19	83

## Data Availability

Data available on request due to restrictions, e.g. privacy or ethical.
